# High heterogeneity undermines generalization of differential expression results in RNA-Seq analysis

**DOI:** 10.1186/s40246-021-00308-5

**Published:** 2021-01-28

**Authors:** Weitong Cui, Huaru Xue, Lei Wei, Jinghua Jin, Xuewen Tian, Qinglu Wang

**Affiliations:** 1 Key Laboratory of Biomedical Engineering & Technology of Shandong High School, Qilu Medical University, Zibo, 255300 China; 2 Environmental Protection Research Institute of Light Industry, Beijing, 100089 China; 3 Shandong Sport University, Jinan, 250102 China

**Keywords:** RNA sequencing, Differential expression, Heterogeneity, Reproducibility, Outlier, Tumor

## Abstract

**Background:**

RNA sequencing (RNA-Seq) has been widely applied in oncology for monitoring transcriptome changes. However, the emerging problem that high variation of gene expression levels caused by tumor heterogeneity may affect the reproducibility of differential expression (DE) results has rarely been studied. Here, we investigated the reproducibility of DE results for any given number of biological replicates between 3 and 24 and explored why a great many differentially expressed genes (DEGs) were not reproducible.

**Results:**

Our findings demonstrate that poor reproducibility of DE results exists not only for small sample sizes, but also for relatively large sample sizes. Quite a few of the DEGs detected are specific to the samples in use, rather than genuinely differentially expressed under different conditions. Poor reproducibility of DE results is mainly caused by high variation of gene expression levels for the same gene in different samples. Even though biological variation may account for much of the high variation of gene expression levels, the effect of outlier count data also needs to be treated seriously, as outlier data severely interfere with DE analysis.

**Conclusions:**

High heterogeneity exists not only in tumor tissue samples of each cancer type studied, but also in normal samples. High heterogeneity leads to poor reproducibility of DEGs, undermining generalization of differential expression results. Therefore, it is necessary to use large sample sizes (at least 10 if possible) in RNA-Seq experimental designs to reduce the impact of biological variability and DE results should be interpreted cautiously unless soundly validated.

**Supplementary Information:**

The online version contains supplementary material available at 10.1186/s40246-021-00308-5.

## Background

RNA-Seq has become an indispensable tool for transcriptome-wide analysis of differential gene expression in oncology to elucidate the mechanism of tumorigenesis and metastasis [1–3]. Due to the high cost [4] and the advantage of low technical variation [5–7] of RNA-Seq technology, many RNA-Seq experiments were performed with very small sample sizes, even with no replicates, but broader biological statements have been drawn on these experiments, discounting the influence of biological variability [8–10].

Extensive genetic intertumoral and intratumoral heterogeneity has long been recognized [11–14]. High genetic heterogeneity may greatly affect differentially expressed gene (DEG) detection in RNA-seq analysis and therefore undermine the reliability of differential expression (DE) results. However, the impact of tumor heterogeneity on the reliability of DE results obtained from RNA-seq data has rarely been studied.

Scientists of a biotechnology firm had tried to confirm published preclinical research findings related to their research, but they were shocked to find that the best-known scientific findings from cancer biology were confirmed in only 6 cases out of 53 [15, 16]. Poor reproducibility of ovarian cancer microRNA profiles has also been reported [17]. The findings above reveal the severity of the reproducibility problem in cancer research, which is probably caused by tumor heterogeneity. As drug development relies heavily on literatures, the problem of irreproducible data may increase the costs of drug development along with the number of late-stage clinical-trial failures [15]. Since RNA-Seq has been used extensively in cancer research, it is urgent to study the potential effect of tumor heterogeneity on the reliability of DE results in RNA-seq analysis.

Normally, it is arduous for researchers to verify their own or other people’s findings due to the difficulty of sampling and limited budget. However, with the help of public large-scale projects which have plenty of samples, such as the Cancer Genome Atlas (TCGA) [18], reproducibility verification of DE results is possible. RNA-Seq data in TCGA database have been extensively employed in studies for understanding genetic changes in tumors [19–23].

In this work, the raw RNA-Seq count data for the three cancer types that have the most samples, namely breast cancer (BRCA), kidney renal clear cell carcinoma (KIRC), and lung adenocarcinoma (LUAD), were obtained from TCGA database. First, we investigated the reproducibility of DE results among the four repeated differential expression analysis, each using totally different samples, for any given number of biological replicates between 3 and 24. Then, we investigated the detection power depending on the number of biological replicates. Finally, we explored why a great many DEGs were not reproducible. All DE analyses were performed using edgeR [24], the most popular R package for DE analysis of RNA-Seq data [9]. The edgeR tool has been proved to have superior specificity and sensitivity as well as good control of false-positive errors [9, 25–27].

## Results

### Number of DEGs depending on the number of biological replicates

**Fig. 1:**
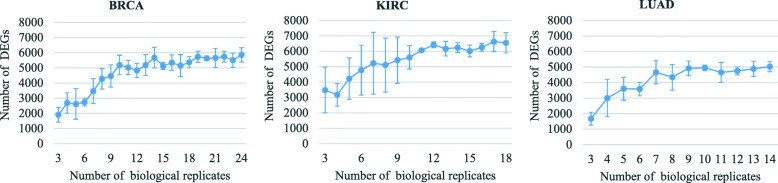
The relationship between the mean number of DEGs and the number of biological replicates. The maximum biological replicate numbers vary depending on the total sample numbers for each cancer type in TCGA. The values represent the *M* ± SD of the number of DEGs for any given number of biological replicates

As shown in Fig. 1, just in terms of quantity, it seems that the more biological replicates used, the more DEGs will be identified. All the three curves in Fig. 1 show an increasing dynamic, but the rate of increase seems to diminish after around 10 biological replicates. It can also be inferred from the error bars that the number of DEGs for a given number of biological replicates generally differs greatly.

### Reproducibility of DE results among the four repeats for a given number of biological replicates

**Fig. 2:**
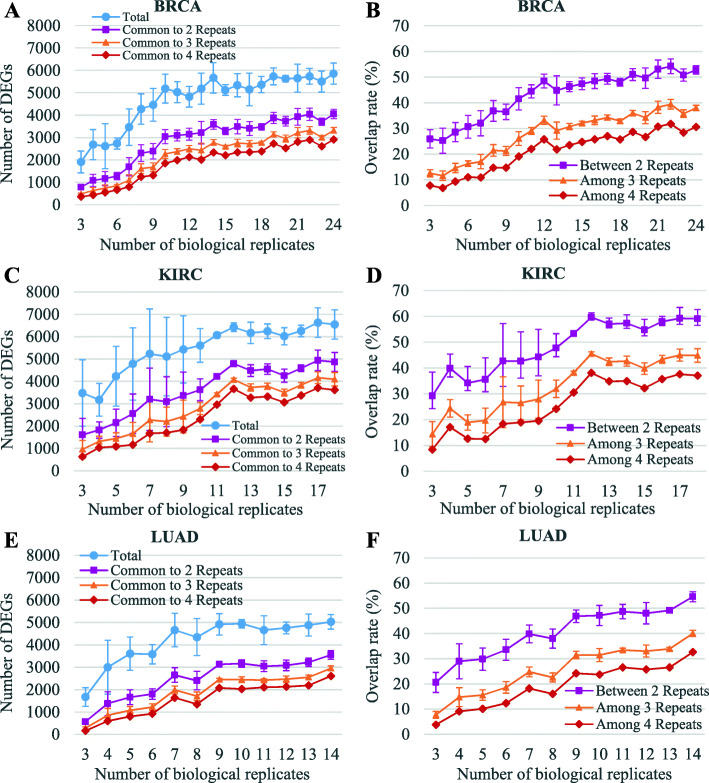
Reproducibility of DE results among the four repeats for a given number of biological replicates. **a**, **c**, **e** The mean number of common DEGs for any two (purple line), three (orange line), or four (red line) repeats for each cancer type depending on the number of biological replicates, and the mean total number of DEGs for any given number of biological replicates (blue line) is also shown for reference. **b**, **d**, **f** The overlap rate of DE results for any two (purple line), three (orange line), or four (red line) repeats for each cancer type depending on the number of biological replicates

As is shown in Fig. 2a, c, and e, for a given number of biological replicates, the number of reproducible DEGs is much less than the mean total number of DEGs, and the more repeats being performed, the lower the number of common DEGs becomes. The results indicate the poor reproducibility of DE results, which can be clearly seen from the changes of overlap rate in Fig. 2b, d, and f as well.

Both the number of common DEGs and the overlap rate increase with the elevated number of biological replicates, but the increasing rate slows down after around 10 biological replicates. For all three cancer types studied, the overlap rates among four repeats are all below 40% for the maximum number of biological replicates, and the percentage drops to below 10% for 3 biological replicates, which implies that the DE results for relatively large sample sizes are not reliable, and the reliability of DE results for small sample sizes are even poorer.

### The evolution of power depending on the number of biological replicates

As it is difficult to choose one repeat to represent the four repeats for any given number of biological replicates, the common DEGs (intersection) and all detected DEGs (union) of the four repeats were used to calculate the power and intersection/union ratio (see the “Materials and methods” section).

**Fig. 3:**
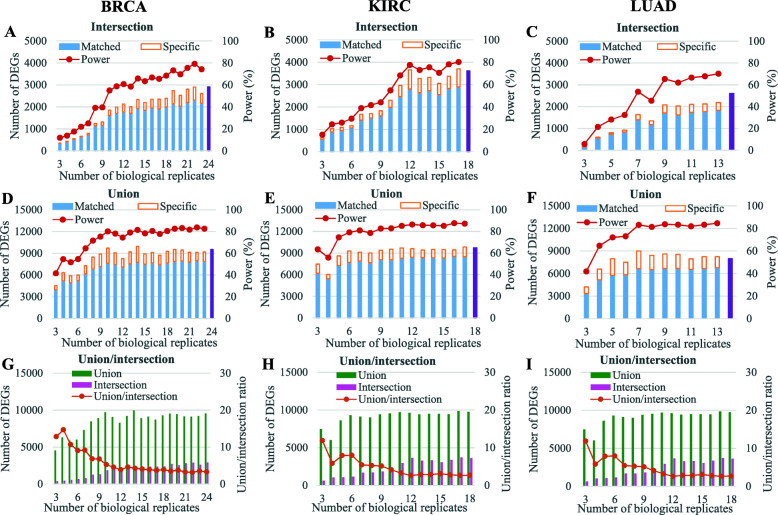
Evolution of detection power and union/intersection depending on the number of biological replicates. **a–c** The number of DEGs and the power of the intersection for a given number of biological replicates for each cancer type. **d**–**f** show the number of DEGs and the power of the union for a given number of biological replicates for each cancer type. For all the stacked bars in charts **a**–**f**, the blue part represents the number of DEGs that match with the corresponding referential intersection or union, while the orange part represents the specific DEGs (not match with the corresponding reference) of the intersection or union. Purple color bars in charts **a**–**f** represent the number of DEGs in the referential intersections or unions for each cancer type. **g**–**i** The number of DEGs in the union (green bar) and intersection (pink bar), as well as the union/intersection ratio (orange line), for a given number of biological replicates for each cancer type

As shown in Fig. 3a, b, and c, for each cancer type, when the number of biological replicates is between 3 and 10, both the number of DEGs and the power of the intersections grow rapidly, but the increasing rate is quite slow after about 10 biological replicates, which is similar to the trend of overlap rate in Fig. 2. As to the unions in Fig. 3d, e, and f, the number of DEGs and the power has similar trends with those of the intersections, but the two indicators reach plateaus faster than those of the intersections do.

The low power of DEG detection for small sample sizes can also be seen from the three curves in Fig. 3a, b, and c. For instance, the power of intersection for 3 biological replicates was below 16% for all three cancer types (as low as 6 % for LUAD), which means that more than 84% of the DEGs in the referential intersection cannot be detected using 3 biological replicates. Our findings clearly reveal that using more biological replicates is not only desirable but needed to improve the DE detection power using RNA-Seq.

As shown in Fig. 3a–f, both the intersection and the union for a given number of biological replicates contain some specific DEGs (i.e., DEGs that do not match with the reference), which means that the DEGs obtained using large sample sizes do not necessarily include all the DEGs obtained using small sample sizes.

As can be seen in Fig. 3g, h, and i, for any given number of biological replicates, the number of DEGs of the union is far larger than that of the intersection, which indicates that most of the DEGs detected in the four repeats for a given number of biological replicates are specific to the samples studied, rather than “true” DEGs for the tumor and normal tissues of each cancer type. This effect is much more intense for small sample sizes, which also reflects the poorer reproducibility of DE results obtained using small sample sizes.

### Dispersion of normalized read counts for non-common DEGs

The results above demonstrate that a large number of DEGs for one repeat are not DEGs for another and these DEGs are referred to as non-common DEGs in this paper. Although non-common DEGs have also been found in previous literatures [9], the cause of the non-common DEGs has rarely been investigated before.

Ten non-common DEGs in repeat II and repeat III for 10 biological replicates in BRCA were used as examples to illustrate the phenomenon, as shown in Table 1. Among the ten genes, *IBSP*, *SGCG*, *DCT*, *APCDD1*, and *DPP6* were identified as DEGs only in repeat III, while *SLC16A3*, *CDH23*, *FOXJ1*, *FGF10*, and *BMP5* were identified as DEGs only in repeat II. The %CV, Log_2_FC, and false discovery rate (FDR) values for the 10 non-common DEGs in KIRC and LUAD are shown in Supplementary Table S1 and S2, respectively.

**Table 1:** Detailed %CV, Log_2_FC, and FDR values for the 10 non-common DEGs in BRCA

Gene symbol	Repeat II				Repeat III			
	%CV		Log_2_FC	FDR	%CV		Log_2_FC	FDR
	N	T			N	T		
*IBSP*	122	167	− 8.14	0.09	115	115	− 6.71	3.80E− 12
*SGCG*	138	241	− 0.32	0.85	234	116	7.38	2.70E− 10
*DCT*	186	307	− 0.49	0.78	152	83	4.79	8.40E− 09
*APCDD1*	70	201	0.57	0.49	45	84	2.57	1.44E− 08
*DPP6*	71	300	− 0.61	0.68	54	143	3.91	4.62E− 08
*SLC16A3*	53	43	− 2.52	3.34E− 09	208	99	− 1.07	0.18
*CDH23*	83	55	2.79	1.12E− 08	91	155	1.38	0.07
*FOXJ1*	59	200	− 5.97	1.82E− 08	207	194	− 1.47	0.16
*FGF10*	63	91	3.27	2.25E− 08	90	178	0.38	0.76
*BMP5*	76	92	4.30	1.41E− 07	93	313	− 2.66	0.08

Capital letters “T” and “N” represent the tumor group and the normal group of each repeat, respectively. The numbers of biological replicates in either tumor groups or normal groups are 10. %CV indicates the percent coefficient of variation

As shown in Table 1, the values of FDR for these genes are all smaller than 2 × 10^−7^ when they are DEGs, which definitely means that they are, statistically, significant DEGs between the tumor and normal group, even if a threshold of 0.0001 is applied to control the FDR. Even so, the ten genes are not identified as DEGs in the other repeat. The result indicates that statistically significant DEGs are not as reliable as commonly believed.

In order to explore the reasons behind the non-common DEGs, we analyzed the dispersion of normalized read counts of these genes for some clues. As shown in Table 1, more than half of the %CVs are above 100. On the whole, there are many more %CVs over 100 in the tumor groups than in the normal groups, with three %CVs in the tumor groups even higher than 300, which probably means that gene expression levels in tumor groups have greater variability than those in normal groups. As CV is the ratio of the standard deviation to the mean, the high %CV reflects great dispersion of normalized read counts. In DE analysis, the high dispersion of read counts for a given gene can cause remarkable changes to the values of log_2_FC and FDR and sometimes may even lead to opposite results. The high variation of expression levels for the same gene in different samples may be the main cause of the poor reproducibility of DE results.

In addition, we noticed in Table 1 that 4 out of the 10 non-common DEGs have opposite regulating trends in different repeats, i.e., upregulated in one repeat, but downregulated in the other, as demonstrated by the values of Log_2_FC. By checking the Log_2_FC values of the 3079 common DEGs between repeat II and III, we found that 35 of them (about 1.14%) also show opposite regulating trends, which indicates that the common DEGs are not reliable either.

**Fig. 4:**
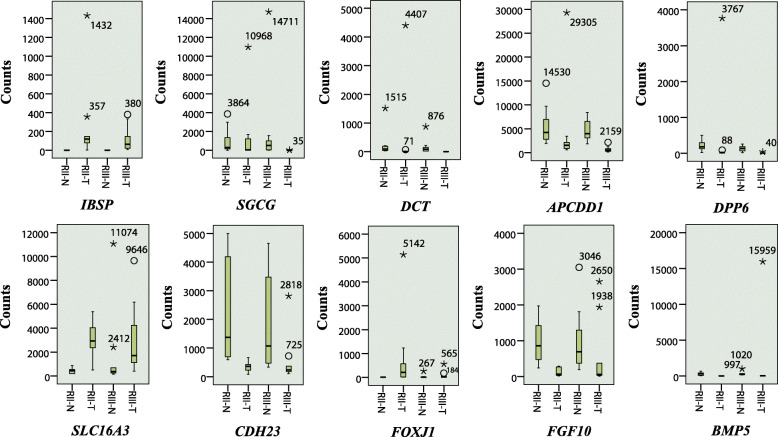
Dispersion of normalized read counts for the 10 non-common genes in BRCA. Mild outliers (more than 1.5 IQR’s from the box, indicated by a circle) and extreme outliers (more than 3 IQR’s from the box, indicated by an asterisk) are shown. The number beside the marker shows the normalized read count value of the point. RII and RIII refer to repeat II and repeat III, respectively. Capital letters “T” and “N” represent the tumor group and the normal group, respectively. IQR indicates the interquartile range

It is clearly shown by the boxplots in Fig. 4 that outlier counts commonly exist in both the tumor and the normal groups, which is also true for the non-common DEGs in KIRC (Supplementary Figure S1) and LUAD (Supplementary Figure S2). Combining the read count dispersion in Fig. 4 with the %CV values in Table 1, we find that the high %CVs are mainly caused by the outlier counts, especially the extreme outliers. By excluding the outlier counts from analysis, 8 out of the 10 non-common DEGs become common DEGs, but the remaining 2 are still non-common DEGs, which implies that the problem of non-common DEGs can be partially resolved by excluding the outliers from analysis. We also confirmed that the opposite regulating trends of the 4 genes described above can be corrected by excluding outlier counts from analysis.

## Discussion

The results above are based on the RNA-Seq data of human tumor and adjacent normal samples. Nevertheless, the issue of low reliability of DEGs for very small sample sizes has also been found in studies using RNA-Seq data from mouse [28, 29], *Saccharomyces cerevisiae* [10], and tomato [9], which implies that the problem of reproducibility is common in RNA-seq analysis.

The maximum number of biological replicates studied here is already much larger than recommended in the literatures [9, 10], but still a large proportion of the DEGs detected are irreproducible. While results obtained using 3 biological replicates for each condition in experimental designs are generally accepted as reliable, our results show quite the opposite, at least in cancer research using RNA-Seq.

As shown in Table 1 and Fig. 4, outlier counts, especially the extremes, account for much of high variation of expression levels. It should be emphasized that outlier counts are commonly scattered in different samples, rather than focused in one sample, in which case the outlier counts can be eliminated by excluding the aberrant sample. As DE analyses with small sample sizes are more susceptible to outliers, the poor reproducibility of DE results for small sample sizes is understandable.

The authenticity of outlier counts is beyond the scope of this research. Nevertheless, figuring out whether the extreme counts are true or not is the prerequisite to properly deal with them. The popular edgeR [24] takes raw read counts as input and provides its own normalization approach [30] but does not handle the outlier counts. Given the enormous influence of outliers on DE analysis, the problem of outlier counts should be properly addressed in future versions of edgeR.

Since low technical variation is one of RNA-Seq’s potential advantages [5–7], most of the variations might be attributed to biological variations which can be reflected in extensive genetic intertumoral and intratumoral heterogeneity [11–14]. Biological variation, unlike measurement error, cannot be reduced with technology improvements, but can only be measured by considering expression measurements taken from multiple biological samples within the same group [8]. Therefore, large sample sizes should be considered when designing RNA-Seq experiments for DGE detection to reduce the effect of biological variability. However, based on our findings, it is impossible to determine an optimal number of biological replicates which can guarantee all detected DEGs are reliable for a given RNA-Seq experiment, but approximately at least 10 replicates per condition should be used to achieve relatively high reproducibility and detection power.

One goal of DE analysis in cancer research by RNA-Seq is to identify and catalog expression of new or alternative transcripts between tumor and normal tissues, which is essential for understanding the mechanism of tumorigenesis and developing effective therapies. Apparently, given the high heterogeneity of tumor and normal samples, it is hard to achieve that goal using small sample sizes, let alone with no biological replicates. Moreover, as demonstrated by our findings, incorporating a relatively larger sample size than recommended for RNA-Seq experiments in previous literatures [9, 27] does not mean the DE results are fully credible.

## Conclusions

In conclusion, both tumor tissue samples and normal tissue samples show high heterogeneity. DE results of small sample sizes are more susceptible to heterogeneity, compared with those of large sample sizes. As a result, reproducibility of DE results and DEG detection power for small sample sizes are far lower than those for large sample sizes. Even if large sample sizes are utilized, a large proportion of the detected DEGs are irreproducible. Therefore, large sample sizes (at least 10 if possible) should be considered in RNA-Seq experimental designs to reduce the interfering effect of sample heterogeneity and DEGs of interest should be validated before making generalized statements.

Similarly, since it is difficult to distinguish which DEGs are specific to the samples in use and which are common to the studied populations, DE results from published RNA-Seq literatures, especially those with very small sample sizes or no biological replicates, should be consulted with caution. With regard to the reproducibility crisis which is particularly severe in cancer biology [15, 16] and has remarkably hindered the translation of cancer research to clinical success [31], much remains to be done to discern the DEGs caused by biological variability and to improve the reproducibility of DE results.

## Materials and methods

### Raw count data collection

Raw RNA-Seq read count data for all available BRCA, KIRC, and LUAD tumors and available adjacent normal tissues were downloaded from The Cancer Genome Atlas (TCGA) database. To ensure sample consistency, data from metastatic or formalin-fixed paraffin-embedded tissue samples [32, 33], as well as repeated data for the same samples, were excluded. After exclusion, total numbers of tumor and normal tissue samples included in BRCA, KIRC, and LUAD datasets were 1177, 610, and 592, respectively.

### DE analysis of the collected raw count data

This work was designed to investigate the evolution of reproducibility of DE results and the detection power depending on the number of biological replicates *n*. Although there are algorithms specially developed for DE analysis of RNA-Seq data without biological replicates [34–36], the results obtained are debatable as it is impossible to estimate the level of biological variability. If there are only two biological replicates, it is difficult to detect an outlier (bad) expression value. Therefore, the minimum *n* was set at 3 for each cancer type.

For each value of *n*, four sets of *n* tumor samples and *n* normal samples were randomly chosen without replacement from datasets of each cancer type to simulate four different experimental repeats, which were denoted as repeat I, II, III, and IV, respectively, for ease of description. For any given *n*, samples in the four repeats were all different. Limited by the number of normal samples in BRCA, KIRC, and LUAD datasets (i.e., 99, 72, and 58, respectively), the maximum *n* for BRCA, KIRC, and LUAD was accordingly set at 24, 18, and 14, respectively. The sampling process was shown in Supplementary Figure S3. Raw read count data of samples in each set of tumor and normal groups were used to construct gene expression matrices for subsequent analyses.

All DE analyses were done with R software (version 3.5.3) and the edgeR package [24] (version 3.22.5). Trimmed-mean *M* values (TMM) normalization was performed to normalize the counts among the different samples [37–40]. As high dispersion of low counts interfered with some of the statistical approximations used in edgeR, genes with low counts were filtered out using the *filterByExpr* function as recommended in the user’s guide. Genes were marked as DEGs if the absolute value of log_2_ transformed fold change (log_2_FC) ≥ 1 and the false discovery rate (FDR) < 0.05.

### Reproducibility of DE results among the four repeats for a given number of biological replicates

As described above, four repeated DE analyses were performed for each number of biological replicates *n*; therefore, four lists of DEGs were obtained for each *n*. To analyze the reproducibility of DE results, we compared the four lists of DEGs in terms of overlap rate which was defined as the ratio of the number of common DEGs (i.e., DEGs that were common to the compared repeats) to the total number of DEGs of the corresponding repeats. For instance, the total number of DEGs identified in repeat II and III for 10 biological replicates in BRCA was 6528, 3079 of which are common to both of the two repeats; therefore, the overlap rate of the DE results for the two repeats was 47.17%. The overlap rate of DE results for any two, three, or four repeats for a given *n* was computed in the same way as exemplified above. Overlap rate was calculated using VENNY (version 2.1.0) (Oliveros, J.C. (2007–2015) Venny; an interactive tool for comparing lists with Venn's diagrams. https://bioinfogp.cnb.csic.es/tools/venny/index.html).

### Power analysis for a given number of biological replicates

We have four lists of DEGs for each *n*, but the number of DEGs and DEG composition of the four lists are quite different, so it is difficult to choose one of the lists as a representative. Therefore, the intersection (i.e., DEGs that are common to all four repeats) and the union (i.e., all DEGs identified for all four repeats) for any given *n* were used for power analysis. Note that the power was defined as the ability for a given sample size to detect “true” DEGs. Obviously, we needed a reference list of “true” DEGs. As is generally accepted that results obtained using larger sample sizes are more robust, the intersection and union of the maximum *n* for each cancer type were used as references.

The power was calculated by the ratio of the number of DEGs in the intersection (or union) for any given *n* to the number of DEGs in the corresponding referential intersection (or union). The ratio of the number of DEGs in the union to that in the intersection for any given *n* was also calculated and marked as union/intersection.

### Read count dispersion analyses for non-common DEGs

The non-common DEGs, as opposed to the common DEGs, were the DEGs that could be identified in one repeat, but not in another. To explore the cause of non-common DEGs, we selected 10 non-common DEGs from the DEG lists of repeat II and repeat III for 10 biological replicates in BRCA dataset and analyzed the characteristics of raw count data of these genes. Although the number of DEGs was close between the two repeats, about 53% of the DEGs were non-common DEGs.

In order to eliminate the interference of different sequencing depth, TMM normalized read counts were used for analysis*.* The percent coefficient of variation (%CV) of normalized read counts in both tumor and normal groups of repeat II and repeat III was calculated for each non-common DEG. Similarly, dispersion of normalized read counts was analyzed and displayed in boxplots using IBM SPSS Statistics (19.0). The read count dispersion analyses for the non-common DEGs in KIRC and LUAD were conducted in the same way as in BRCA.

## Supplementary Information

**Additional file 1: Supplementary Table S1.** Detailed %CV, Log_2_FC, and FDR values for the 10 non-common DEGs in KIRC

**Additional file 2: Supplementary Table S2.** Detailed %CV, Log_2_FC, and FDR values for the 10 non-common DEGs in LUAD

**Additional file 3: Supplementary Figure S1.** Dispersion of normalized read counts for the 10 non-common genes in KIRC. Mild outliers (more than 1.5 IQR’s from the box, indicated by O) and extreme outliers (more than 3 IQR’s from the box, indicated by *) are shown. The number beside the marker shows the normalized count value of the point. RII and RIII refer to repeat II and repeat III, respectively. Capital letters “T” and “N” represent the tumor group and the normal group, respectively. IQR indicates the interquartile range.

**Additional file 4: Supplementary Figure S2.** Dispersion of normalized read counts for the 10 non-common genes in LUAD. Mild outliers (more than 1.5 IQR’s from the box, indicated by O) and extreme outliers (more than 3 IQR’s from the box, indicated by *) are shown. The number beside the marker shows the normalized count value of the point. RII and RIII refer to repeat II and repeat III, respectively. Capital letters “T” and “N” represent the tumor group and the normal group, respectively. IQR indicates the interquartile range.

**Additional file 5: Supplementary Figure S3.** Diagram of experimental design for the BRCA dataset. The processes of sampling and analysis for the KIRC and LUAD datasets were the same as that of BRCA. The numbers of tumor tissue samples and adjacent normal tissue samples for KIRC were 526 and 72, respectively, while the two numbers were 509 and 58, respectively, for LUAD. Restrained by the number of normal tissue samples which was far less than the number of tumor samples for each cancer type, the maximum number of biological replicates for BRCA, KIRC, and LUAD was accordingly set at 24, 18, and 14, respectively.

## Data Availability

The datasets supporting the conclusions of this article are available in TCGA repository, https://www.cancer.gov/tcga.

## Abbreviations

BRCA: Breast cancer
CV: Coefficient of variation
DE: Differential expression
DEGs: Differentially expressed genes
FC: Fold change
FDR: False discovery rate
IQR: Interquartile range
KIRC: Kidney renal clear cell carcinoma
LUAD: Lung adenocarcinoma
RNA-seq: RNA sequencing
SD: Standard deviation
TCGA: The Cancer Genome Atlas
TMM: Trimmed-mean *M* values
