# Supplementary material for: High heterogeneity undermines generalization of differential expression results in RNA-Seq analysis
Source: Hum Genomics. 2021 Jan 28;15:7. doi: 10.1186/s40246-021-00308-5 (PMC7845028; doi:10.1186/s40246-021-00308-5)
Supplement: Supplementary file 1 — Additional file 1: Supplementary Table S1. Detailed %CV, Log2FC, and FDR values for the 10 non-common DEGs in KIRC [file 40246_2021_308_MOESM1_ESM.docx]

**Supplementary Table S1** **Detailed %CV, Log_2_FC, and FDR values for the 10** **non-common DEGs in KIRC**

| Gene symbol | Repeat II | | | |  | Repeat III | | | |
| --- | --- | --- | --- | --- | --- | --- | --- | --- | --- |
|  | %CV | | Log_2_FC | FDR |  | %CV | | Log_2_FC | FDR |
|  | N | T |  |  |  | N | T |  |  |
| *CKMT2* | 40.80 | 77.28 | 3.59 | 5.53E-18 |  | 67.36 | 199.62 | 0.19 | 0.86 |
| *NAT2* | 81.60 | 55.53 | 3.79 | 5.95E-16 |  | 80.54 | 114.62 | 1.06 | 0.13 |
| *DIRAS1* | 64.15 | 102.62 | 4.55 | 9.51E-15 |  | 70.95 | 178.96 | 1.32 | 0.15 |
| *MCM10* | 64.41 | 39.59 | -3.68 | 1.25E-15 |  | 230.81 | 63.28 | -1.17 | 0.14 |
| *TPX2* | 55.34 | 45.88 | -3.07 | 1.39E-14 |  | 196.43 | 62.81 | -1.21 | 0.09 |
| *ANKRD22* | 64.03 | 198.51 | -0.09 | 0.93 |  | 65.45 | 40.74 | 2.48 | 8.42E-09 |
| *RPL10P9* | 216.69 | 167.16 | -0.39 | 0.73 |  | 60.50 | 136.34 | -4.10 | 8.59E-08 |
| *WT1-AS* | 69.78 | 229.08 | 1.83 | 0.07 |  | 90.57 | 157.60 | 4.36 | 7.98E-07 |
| *MTDHP3* | 117.38 | 308.20 | 2.17 | 0.14 |  | 143.17 | 122.34 | 3.97 | 2.46E-06 |
| *NUPR1* | 53.66 | 58.36 | -0.49 | 0.20 |  | 73.78 | 82.17 | -2.23 | 6.27E-06 |

Capital letters “T” and “N” represent the tumor group and the normal group of each repeat, respectively. The numbers of biological replicates in either tumor groups or normal groups are 10. %CV indicates the percent coefficient of variation.
