# Supplementary figures and images for: High heterogeneity undermines generalization of differential expression results in RNA-Seq analysis
Source: Hum Genomics. 2021 Jan 28;15:7. doi: 10.1186/s40246-021-00308-5 (PMC7845028; doi:10.1186/s40246-021-00308-5)

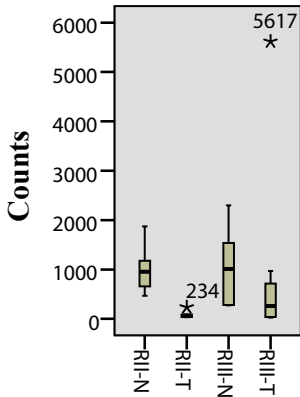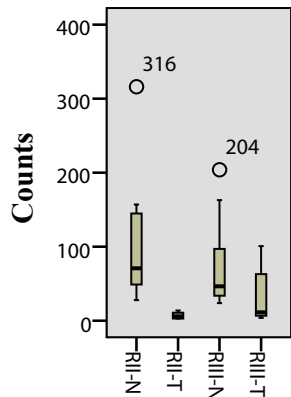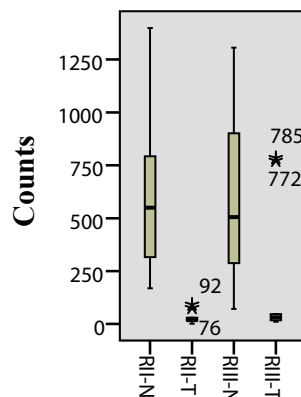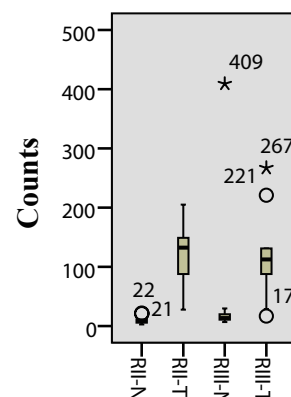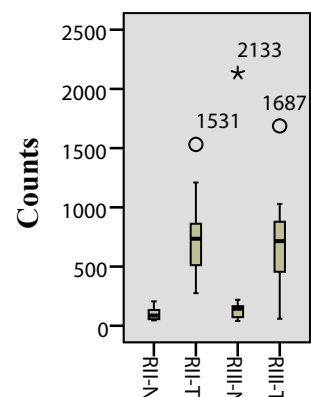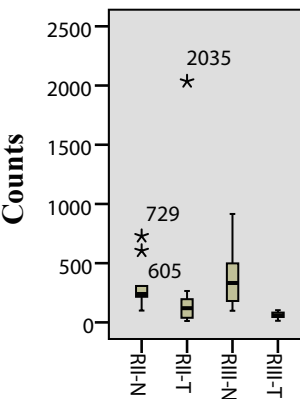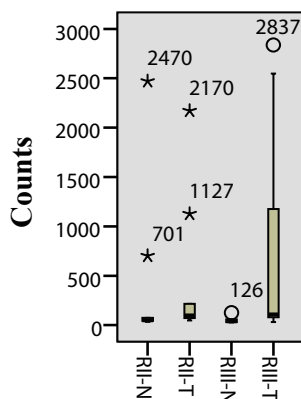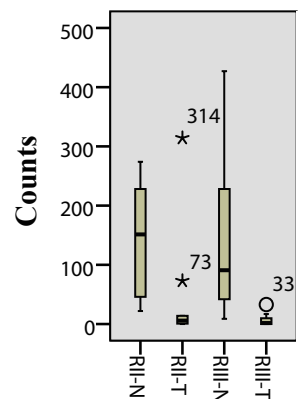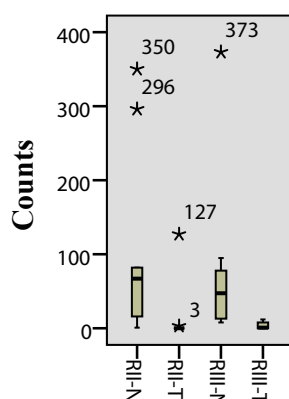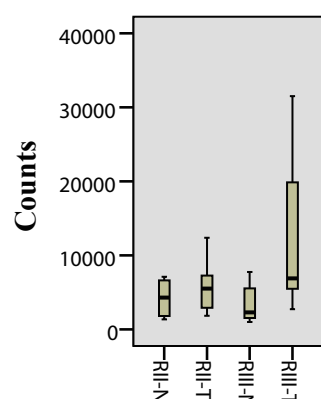

Supplement: Supplementary file 3 — Additional file 3: Supplementary Figure S1. Dispersion of normalized read counts for the 10 non-common genes in KIRC. Mild outliers (more than 1.5 IQR’s from the box, indicated by O) and extreme outliers (more than 3 IQR’s from the box, indicated by *) are shown. The number beside the marker shows the normalized count value of the point. RII and RIII refer to repeat II and repeat III, respectively. Capital letters “T” and “N” represent the tumor group and the normal group, respectively. IQR indicates the interquartile range. [file 40246_2021_308_MOESM3_ESM.pdf]

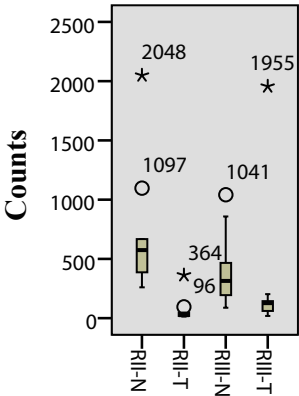

*BCHE*

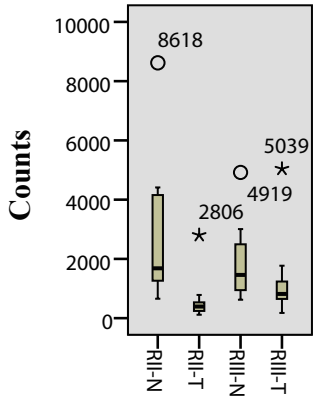

*VGLL3*

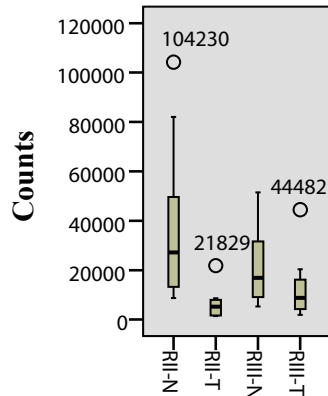

*CCN2*

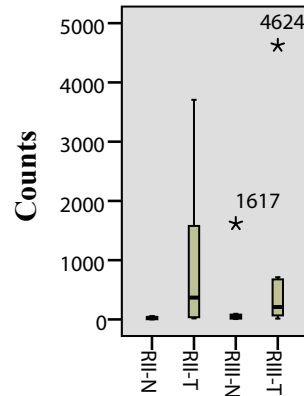

*IGLV3\_9*

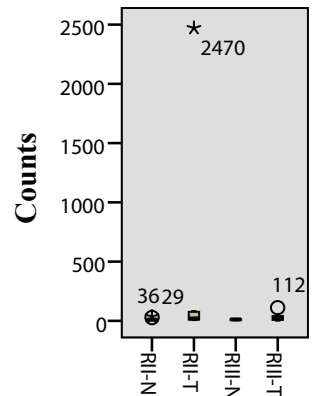

*DPF1*

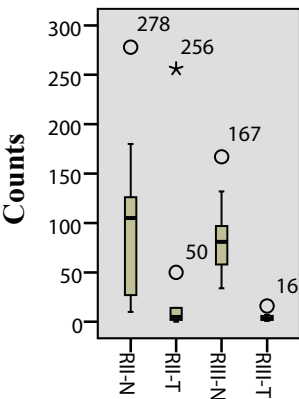

*TRIM58*

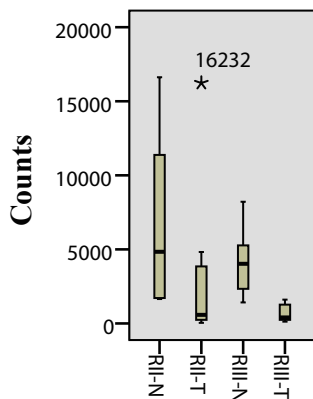

*GPC3*

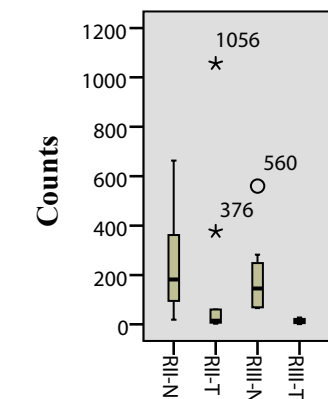

*MEGF11*

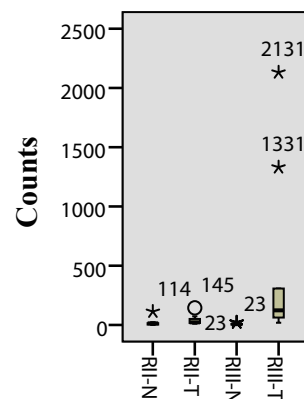

*NXPH4*

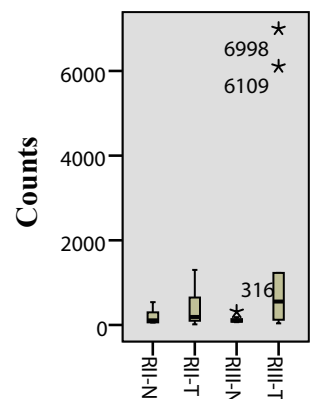

*SYT1*

Supplement: Supplementary file 4 — Additional file 4: Supplementary Figure S2. Dispersion of normalized read counts for the 10 non-common genes in LUAD. Mild outliers (more than 1.5 IQR’s from the box, indicated by O) and extreme outliers (more than 3 IQR’s from the box, indicated by *) are shown. The number beside the marker shows the normalized count value of the point. RII and RIII refer to repeat II and repeat III, respectively. Capital letters “T” and “N” represent the tumor group and the normal group, respectively. IQR indicates the interquartile range. [file 40246_2021_308_MOESM4_ESM.pdf]
