# Supplementary material for: High heterogeneity undermines generalization of differential expression results in RNA-Seq analysis
Source: Hum Genomics. 2021 Jan 28;15:7. doi: 10.1186/s40246-021-00308-5 (PMC7845028; doi:10.1186/s40246-021-00308-5)
Supplement: Supplementary file 5 — Additional file 5: Supplementary Figure S3. Diagram of experimental design for the BRCA dataset. The processes of sampling and analysis for the KIRC and LUAD datasets were the same as that of BRCA. The numbers of tumor tissue samples and adjacent normal tissue samples for KIRC were 526 and 72, respectively, while the two numbers were 509 and 58, respectively, for LUAD. Restrained by the number of normal tissue samples which was far less than the number of tumor samples for each cancer type, the maximum number of biological replicates for BRCA, KIRC, and LUAD was accordingly set at 24, 18, and 14, respectively. [file 40246_2021_308_MOESM5_ESM.pdf]

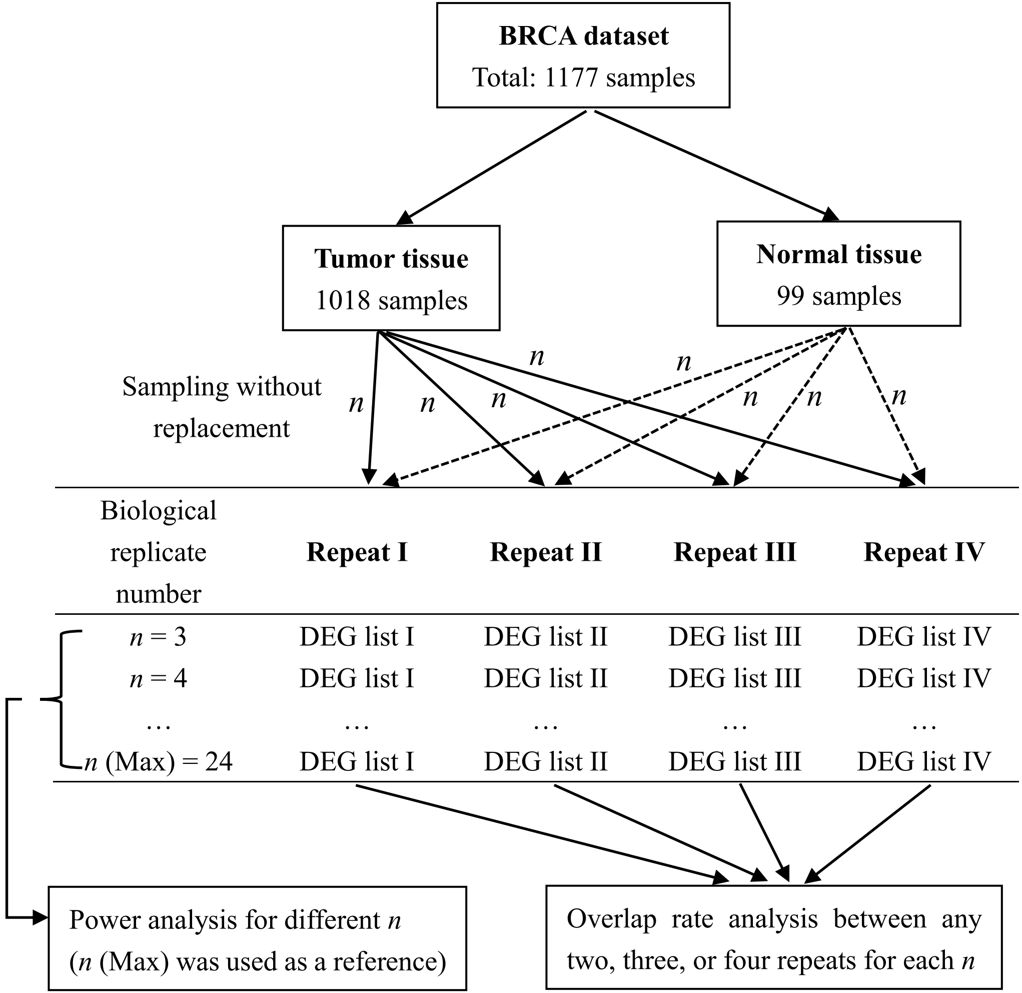
BRCA dataset
Total: 1177 samples
Tumor tissue
1018 samples
Normal tissue
99 samples
Sampling without replacement
n
Biological replicate number
Repeat I
Repeat II
Repeat III
Repeat IV
n = 3
n = 4
n (Max) = 24
DEG list I
DEG list II
DEG list III
DEG list IV
Power analysis for different n (n (Max) was used as a reference)
Overlap rate analysis between any two, three, or four repeats for each n
